# Editorial Comment: Penile prosthesis implant in the special populations: diabetics, neurogenic conditions, fibrotic cases, concurrent urinary continence surgery, and salvage implants

**DOI:** 10.1590/S1677-5538.IBJU.2020.03.04

**Published:** 2020-02-20

**Authors:** E Chung, Valter Javaroni

**Affiliations:** 1 AndroUrology Centre BrisbaneQLD Australia AndroUrology Centre, Brisbane, QLD 4000, Australia; 2 University of Queensland Princess Alexandra Hospital BrisbaneQLD Australia University of Queensland, Princess Alexandra Hospital, Brisbane, QLD 4000, Australia; 3 Macquarie University Hospital SydneyNSW Australia Macquarie University Hospital, Sydney, NSW 2109, Australia; 1 Hospital Federal do Andaraí Rio de Janeiro Departamento de Andrologia Rio de JaneiroRJ Brasil Departamento de Andrologia, Hospital Federal do Andaraí Rio de Janeiro, Rio de Janeiro, RJ, Brasil

## COMMENT

In this invited review Dr. Chung from Australia presented a good revision on Penile Prosthesis Implant (PPI) pointing to important technical aspects about this therapeutic option considering special populations.

The author conducted a critical review of all relevant publications from Medline and Embase databases and included a brief review of surgical challenges and a practical action-based set of recommendations on surgical options.

The risk of infection is still a matter and in diabetics it is more frequent and serious. Dr. Chung presented controversial numbers on ideal cut off value on HbA1c level and the lack of an evidence-based guideline published that precludes surgery above a certain value for HbA1c. He also stressed on the protective effect of antibiotic-impregnated implants (1) and high-volume surgeons (2). Postoperatively, diet and usual diabetes medications should be restarted as soon as possible, often under the guidance of a multidisciplinary team.

Other important special group is the one formed by spinal cord injury (SCI) men. Besides the fact that literature on the clinical outcomes of PPI surgery among neurogenic men is limited, Dr. Chung presented the question of which would be the best option for SCI patients: inflatable three-piece or the malleable or semi-rigid prosthesis, since they are often physically handicapped with poor hand dexterity, limited range of mobility, and muscle fatigue. On the other hand, the lack of sensation among SCI men may predispose those with a malleable implant to have a delayed identification and presentation of prosthetic complications (3). Discussion on the advantages and disadvantages between malleable and inflatable PPI should be conducted based on the patient's physical characteristics, sexual needs, and cost.

The author also approached corporal fibrosis (e.g., Peyronie's disease or priapism), and salvage PPI surgery, since those cases can pose a substantial technical challenge in terms of corporal dilation and they do have a greater risk of prosthetic complications, especially device infection and erosion.
